# Epidemiological Surveillance of Parvoviruses in Commercial Chicken and Turkey Farms in Guangxi, Southern China, During 2014–2019

**DOI:** 10.3389/fvets.2020.561371

**Published:** 2020-10-08

**Authors:** Yanfang Zhang, Bin Feng, Zhixun Xie, Xianwen Deng, Minxiu Zhang, Zhiqin Xie, Liji Xie, Qing Fan, Sisi Luo, Tingting Zeng, Jiaoling Huang, Sheng Wang

**Affiliations:** Guangxi Key Laboratory of Veterinary Biotechnology, Guangxi Veterinary Research Institute, Nanning, China

**Keywords:** parvovirus, chicken parvovirus, turkey parvovirus, epidemiological surveillance, Southern China

## Abstract

A previously unidentified chicken parvovirus (ChPV) and turkey parvovirus (TuPV) strain, associated with runting-stunting syndrome (RSS) and poultry enteritis and mortality syndrome (PEMS) in turkeys, is now prevalent among chickens in China. In this study, a large-scale surveillance of parvoviruses in chickens and turkeys using conserved PCR assays was performed. We assessed the prevalence of ChPV/TuPV in commercial chicken and turkey farms in China between 2014 and 2019. Parvoviruses were prevalent in 51.73% (1,795/3,470) of commercial chicken and turkey farms in Guangxi, China. The highest frequency of ChPV positive samples tested by PCR occurred in chickens that were broiler chickens 64.18% (1,041/1,622) compared with breeder chickens 38.75% (572/1,476) and layer hens 38.89% (112/288), and TuPV was detected in 70/84 (83.33%). Native and exotic chicken species were both prevalent in commercial farms in southern China, and exotic broiler chickens had a higher positive rate with 88.10% (148/168), while native chickens were 50.00% (1,465/2,930). The environmental samples from poultry houses tested positive for ChPV and TuPV were 47.05% (415/874). Samples from open house flocks had higher prevalence rates of ChPV than those of closed house flocks (**Table 5**), among which those from the open house showed 84.16% (85/101) positivity, those from litter showed 62.86% (44/70) positivity, and those from drinking water showed 50.00% (56/112) positivity, whereas those from the closed house litter were 53.57% (60/112), those from swabs were 50.18% (138/275), and those from drinking water were 15.69% (32/204). Samples collected during spring were more frequently ChPV/ TuPV positive than those collected during other seasons. This study is the first report regarding the epidemiological surveillance of ChPV and TuPV in chicken/turkey flocks in Guangxi, China. Our results suggest that ChPV and TuPV are widely distributed in commercial fowl in Guangxi. These findings highlight the need for further epidemiological and genetic research on ChPV and TuPV in this area.

## Introduction

Vertebrates, both animals and humans can be infected with small, non-enveloped parvoviruses. The genomes of these vertebrate parvoviruses are ~5 kb in size, and are classified in the subfamily Parvoviridae, including the genus *Parvovirus* (1). These viruses have been linked to gastrointestinal diseases in human and other mammals (2–4). Avian parvovirus is one of the most important pathogens causing intestinal diseases in poultry (5). Chicken parvovirus (ChPV) was detected by electron microscopy for the first time (6, 7). Since then, ChPV have been identified as the cause of runting and stunting syndrome (RSS) in chickens, which is characterized by significant growth retardation with poor feather development and bone disease (7). Turkey parvovirus (TuPV) has been reported by Trampel et al. (8), presenting as poult enteritis and mortality syndrome (PEMS) in turkeys, and is considered responsible for the incidence of intestinal diseases and increase in the mortality rate of sick birds. Also, it has been detected in Derzsy's disease in goslings and Muscovy ducks (5, 6, 8, 9).

A non-structural gene (NS) and a structural viral protein (VP) gene (10) are two major genes of parvoviruses. The VP gene is located at the 3′ end; the NS gene is at the 5′ end, which encodes a small number of replication proteins and appears to be a highly conserved region (10, 11). ChPV and TuPV genome sequence analysis showed strong similarity between the two, although they are less closely phylogenetically related to geese parvoviruses and Muscovy ducks parvoviruses (12, 13). Attempts to isolate ChPV and TuPV in tissue cultures or embryonated eggs have remained unsuccessful, except for in a study conducted in Brazil (14).

Economic losses due to increased RSS and PEMS have become a continual worldwide problem that influences the development of the poultry industry. RSS and PEMS are characterized by diarrhea, anorexia, malabsorption, stunting, and poor feed conversion, which lead to immunosuppression (3). Studies have showed that many viruses have been associated with RSS and PEMS, such as reovirus, coronavirus, astrovirus, rotavirus, and parvoviruses (3, 15–17). However, none of these viruses has been proved to be the only cause of RSS and PEMS. In addition to a lack of a clear understanding of the cause, vaccines have not been developed for these syndromes. These viruses have been detected and isolated in healthy and diseased birds, which indicates that interaction occurs between the virus and unidentified additional agents (18).

China is mainly based on commercial large-scale chicken farms, and products from commercial poultry farm are an important source of protein in the Chinese population, and ChPV has been a restrictive factor in commercial poultry farms. ChPV shedders among healthy flocks could be one of the main causes of the epidemiological factor in this disease. ChPV/TuPV is emerging and re-emerging worldwide (14, 19–23). However, research studies on molecular detection and epidemiologic investigations of ChPV/TuPV in China are rarely conducted. The infection statuses of the ChPV/TuPV strains in Guangxi are unknown; thus, we conducted this study to investigate the epidemiology of ChPV and TuPV in Guangxi poultry flocks. Our preliminary findings indicate that newly emerged ChPV/TuPV variants can be detected in commercial poultry in Guangxi, China.

This study could contribute to the design and development of effective disease prevention and control strategies to reduce economic losses due to emerging viruses.

## Materials and Methods

### Sample Collection for Surveillance

Epidemiological surveillance of ChPV/TuPV in poultry was conducted at 80 randomly selected commercial chicken and turkey farms in Guangxi from October 2014 to November 2019 (**Table 2**). Each farm was visited once, and ~12 to 120 birds were sampled in each poultry house; 10–30 individual cloacal swabs were collected from each flock. Cloacal samples were collected from 227 commercial chicken (genus *Gallus*) flocks and 6 commercial turkey flocks.

Exotic chickens were imported grandparent stock of specific strains of western chicken breeds, and native chickens were primary breeder stock of miscellaneous breeds that have been kept for several generations in China. Exotic and Chinese native chickens, which included broilers, layers and breeders, were reared intensively at commercial farms. Samples were collected from broiler chickens 1–21 wks old, breeders 1–54 wks old and layer hens 3–60 wks old.

Biological samples were collected from the cloacae of both healthy and diseased chickens and turkeys using cotton swabs. The environmental samples were collected at different points in each poultry house, including litter samples, water samples, and swab samples collected from the walls, floors, feed pads, and drinkers (**Table 2**).

According to the World Organization for Animal Health (OIE) protocol (https://www.oie.int/fileadmin/Home/eng/Health_standards/tahm/1.01.02_COLLECTION_DIAG_SPECIMENS.pdf), all the samples including cloacal swabs and environmental swabs were collected and stored separately in 1.5 mL of storage medium transported in a 2.0 mL Eppendorf tube (EP) on ice until processing. The storage medium contained 10,000 U/mL of penicillin and 10 mg/mL of streptomyin in sterile PBS (phosphate-buffered saline).

### DNA Extraction

The cloacal swabs and environmental suspensions were homogenized and centrifuged at 6,000 g for 5 min. In accordance with the manufacturer's instructions for commercial kits (Transgen, Beijing, China), 200 μL of each supernatant was used for DNA extraction.

### PCR Detection

(1) PCR using a set of specific primers (NS561F and NS561R) targeting the ChPV/TuPV NS gene and amplifying a 561 bp (16) fragment, and (2) nested PCR (nPCR) using 2 sets of specific primers (VP1 and VP2 for the first round and VP3 and VP4 for the second round) targeting the ChPV/TuPV VP gene and amplifying a 249 bp (24) fragment were carried out to determine whether the samples contained to ChPV/TuPV. The PCR and nPCR primers information is shown in Table 1.

**Table 1:** Primer information.

**Primer name**	**Primer sequence (5^**′**^ → 3^**′**^)**	**Product size (bp)**	**Annealing temperature (°C)**
NS561F	TTCTAATAACGATATCACTCAAGTTTC	561	55
NS561R	TTTGCGCTTGCGGTGAAGTCTGGCTCG		
VP1	TGGAATTGTGATACTATATGGG	373	56
VP2	TCYTGATCTGCAAATATTTG		
VP3	CATTGTGTCTGTCTWATGCGTGAC	249	64
VP4	GTTTTCTGGATGACTTGCA		

The PCR assays were conducted as described in published procedures (16). The nPCRs targeting the VP1/VP2 regions were prepared in the same way, except the annealing temperature reached 56/64°C separately (16, 24). The PCR products were visualized by 1.2% agarose gel electrophoresis.

## Results

### PCR Detection for Commercial Chicken and Turkey Flocks

**Figure 1:**
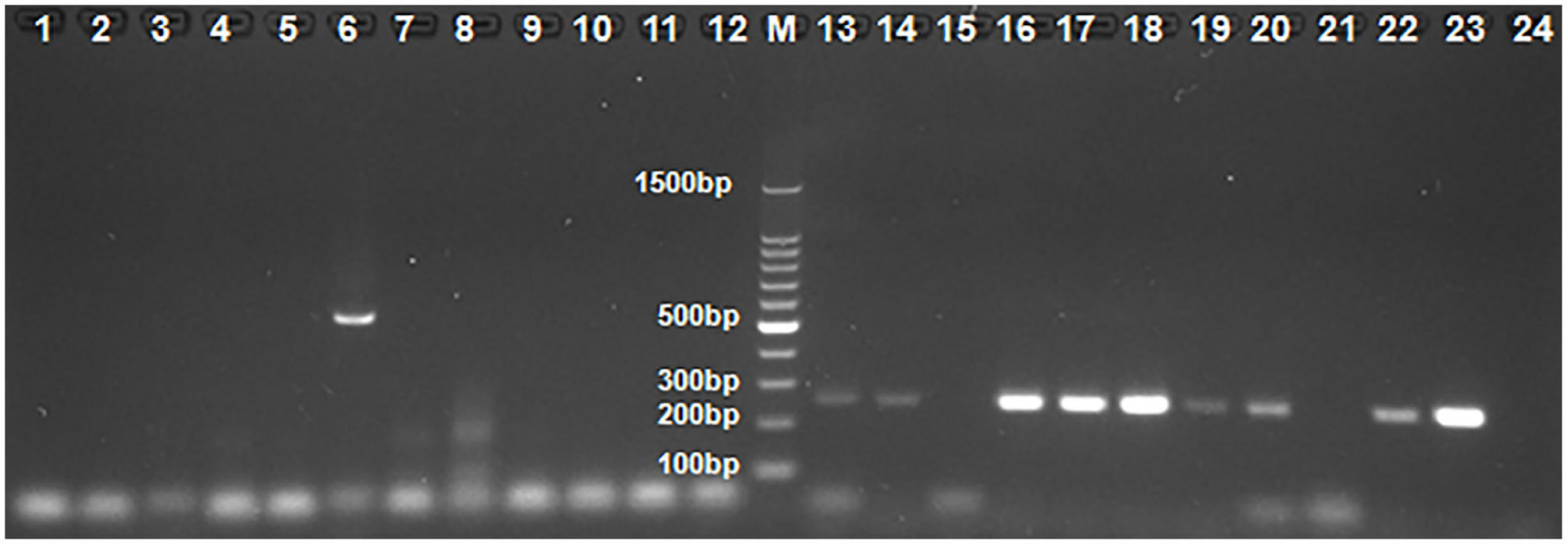
The results by some clinical samples used PCR and nested PCR for ChPV/TuPV detection. M: 100-bp DNA ladder marker; 1–12 and 13–24: 12 Clinical sample of No. 23 farm used PCR and nested PCR for ChPV/TuPV detection, respectively; 6: ChPV/TuPV NS 561 bp product; 13, 14, 16, 17, 18, 19, 20, 22, 23: ChPV/TuPV VP 239 bp product.

Among the 3,470 tested animals, 283 chickens and 12 turkeys were culled because of exhibiting diarrhea, poor weight gain, malabsorption syndrome, and mortality. Between 2014 and 2019, we collected 3,470 swabs from 233 flocks in 80 commercial poultry farms, and 51.73% (1,795/3,470) were PCR-positive for ChPV/TuPV, including 50.95% of commercial chicken farms and 83.33% of commercial turkey farms (Figure 1, Table 2). Compared to in turkeys, natural parvovirus infection was more frequently detected in chickens. However, TuPV was more prevalent than ChPV in the tested flocks. PCR assays showed that there were negative results in one of six examined turkey flocks. These data were similar to the reported 77–78% infection rates in the United States commercial chicken and turkey flocks in a survey conducted between 2003 and 2008 (16). The presence of ChPV/TuPV prevalence in Hungarian and Croatian commercial poultry flocks was also reported, but the infection rates in those countries were unknown (19, 20).

**Table 2:** ChPV/TuPV detection and information on clinical samples.

**No. farms**	**Type**	**No. flocks**	**No. of the swabs**	**ChPV/TuPV PCR (%)**		**Environment samples (%)**		**No. of RSS-like (%)**
				**NS1 (PCR)**	**VP1/VP2 (nested PCR)**	**Close house**	**Open house**	
2014-1	A	4	60	14 (23.33)	30 (50.00)	–	–	–
2014-2	B	2	60	6 (10.00)	23 (38.33)	–	–	–
2014-3	B	7	84	28 (33.33)	40 (47.62)	–	–	4/5 (80.00)
	C	3	36	2 (5.56)	2 (5.56)	–	–	–
2014-4	B	6	48	5 (10.42)	21 (43.75)	10/12 (83.33)	–	–
2014-5	B	3	48	35 (72.92)	47 (97.92)	8/12 (66.67)	–	–
2014-6	C	7	84	59 (70.24)	74 (88.10)	0/2 (0.00)	6/7 (85.71)	18/20 (90.00)
2014-7	B	6	72	32 (53.33)	39 (54.17)	10/15 (66.67)	1/3 (33.33)	–
2015-8	B	5	60	24 (40.00)	41 (68.33)	6/12 (50.00)	–	–
2015-9	C	5	60	33 (55.00)	40 (66.67)	2/4 (50.00)	5/8 (62.50)	10/12 (83.33)
2015-10	C	6	90	50 (55.56)	80 (88.89)	5/8 (62.50)	10/16 (62.50)	14/16 (87.5)
2015-11	D	1	12	2 (16.67)	0 (0.00)	0/2 (0.00)	0/4 (0.00)	–
2015-12	D	2	24	22 (91.67)	24 (100)	2/4 (50.00)	8/8 (100.00)	11/12 (91.67)
2015-13	C	4	48	4 (8.33)	26 (54.17)	2/4 (50.00)	6/8 (75.00)	8/8 (100.00)
2015-14	B	4	48	24 (50.00)	32 (66.67)	4/12 (33.33)	–	–
2015-15	D	3	48	34 (70.83)	46 (95.83)	3/4 (75.00)	5/8 (62.50)	–
2015-16	B	3	36	0 (0.00)	9 (25.00)	2/10 (20.00)	–	–
2015-17	C	2	48	44 (91.67)	46 (95.83)	0/2 (0.00)	5/8 (62.50)	10/10 (100.00)
2015-18	B	4	48	12 (25.00)	30 (62.50)	12/12 (100)	–	–
2015-19	B	4	48	21 (43.75)	25 (52.08)	5/12 (41.67)	–	–
2015-20	E	4	48	32 (66.67)	41 (85.42)	2/4 (50.00)	6/8 (75.00)	11/12 (91.67)
2015-21	E	2	24	3 (12.50)	12 (50.00)	2/12 (16.67)	–	–
2015-22	E	4	48	44 (91.67)	47 (97.92)	1/2 (50.00)	6/7 (85.71)	9/10 (90.00)
2015-23	A	5	60	1 (1.67)	10 (16.67)	12/15 (80.00)	4/5 (80.00)	–
2015-24	A	4	48	2 (4.17)	16 (33.33)	0/12 (0.00)	–	–
2015-25	B	2	20	7 (35.00)	7 (35.00)	10/30 (33.33)	1/4 (25.00)	–
2015-26	E	4	48	40 (83.33)	48 (100)	2/4 (50.00)	3/8 (37.50)	11/12 (91.67)
2015-27	C	2	24	4 (16.67)	11 (45.83)	–	–	4/4 (100.00)
2016-28	C	3	24	8 (33.33)	16 (66.67)	–	–	3/4 (75.00)
2016-29	C	3	24	4 (16.67)	14 (58.33)	–	–	4/4 (100.00)
2016-30	C	4	48	23 (47.92)	33 (68.75)	–	–	10/12 (83.33)
2017-31	B	4	48	24 (50.00)	22 (45.83)	4/12 (33.33)	–	–
2017-32	C	4	40	9 (22.50)	22 (55.00)	2/2 (100.00)	6/8 (75.00)	9/10 (90.00)
2017-33	C	2	20	4 (20.00)	10 (50.00)	–	–	2/2 (100.00)
2017-34	C	2	20	5 (25.00)	12 (60.00)	–	–	5/5 (100.00)
2017-35	B	4	48	0 (0.00)	4 (8.33)	2/12 (16.67)	–	–
2017-36	C	4	48	13 (27.08)	40 (83.33)	2/4 (50.00)	8/8 (100.00)	10/12 (83.33)
2017-37	B	4	48	0 (0.00)	1 (2.08)	1/12 (8.33)	–	–
2017-38	C	4	48	3 (6.25)	26 (54.17)	2/4 (25.00)	7/8 (100.00)	8/8 (100.00)
2017-39	C	4	48	28 (58.33)	39 (81.25)	1/4 (50.00)	6/8 (75.00)	10/10 (100.00)
2017-40	B	4	48	13 (27.08)	37 (77.08)	9/12 (75.00)	–	–
2017-41	B	4	48	11 (22.92)	17 (35.42)	5/12 (41.67)	–	–
2018-42	B	3	60	21 (35.00)	57 (95.00)	11/12 (91.67)	–	–
2018-43	B	3	60	0 (0.00)	6 (10.00)	–	–	–
2018-44	C	1	20	20 (100.00)	20 (100.00)	4/6 (66.67)	6/6 (100.00)	3/3 (100.00)
2018-45	C	1	20	20 (100.00)	20 (100.00)	3/3 (100.00)	5/5 (100.00)	3/4 (75.00)
2018-46	C	2	40	10 (25.00)	12 (30.00)	0/6 (0.00)	3/10 (30.00)	4/6 (66.67)
2018-47	C	2	40	17 (42.50)	19 (47.50)	1/8 (12.50)	3/8 (37.50)	5/6 (83.33)
2018-48	C	6	120	33 (27.50)	66 (55.00)	3/10 (30.00)	9/12 (75.00)	6/12 (50.00)
2018-49	C	1	20	0 (0.00)	3 (15.00)	0/6 (0.00)	1/6 (16.67)	–
2018-50	C	1	20	8 (40.00)	8 (40.00)	0/6 (0.00)	2/6 (33.33)	–
2018-51	B	2	40	0 (0.00)	1 (2.50)	1/12 (8.33)	–	–
2018-52	B	2	40	39 (97.50)	39 (97.50)	8/12 (66.67)	–	–
2018-53	B	1	20	9 (45.00)	9 (45.00)	–	–	–
2018-54	B	1	20	0 (0.00)	0 (0.00)	0/12 (0.00)	–	–
2018-55	C	2	40	9 (22.50)	9 (22.50)	0/6 (0.00)	1/6 (16.67)	–
2018-56	C	1	20	20 (100.00)	20 (100.00)	4/4 (100.00)	4/4 (100.00)	6/6 (100.00)
2018-57	C	1	20	14 (70.00)	16 (80.00)	1/2 (50.00)	1/2 (50.00)	4/4 (100.00)
2018-58	C	1	20	3 (15.00)	3 (15.00)	–	–	–
2018-59	C	1	20	20 (100.00)	20 (100.00)	6/6 (100.00)	6/6 (100.00)	6/6 (100.00)
2018-60	A	1	20	10 (50.00)	10 (50.00)	5/12 (41.67)	–	–
2018-61	A	2	40	15 (37.50)	17 (42.50)	10/20 (50.00)	–	–
2018-62	A	2	40	12 (30.00)	13 (32.50)	3/12 (25.00)	–	–
2018-63	A	1	20	16 (80.00)	16 (80.00)	8/12 (80.00)	–	–
2019-64	C	3	60	32 (53.33)	37 (61.67)	2/16 (12.50)	10/20 (50.00)	4/8 (50.00)
2019-65	B	6	120	2 (1.67)	3 (2.50)	2/12 (16.67)	–	–
2019-66	C	1	20	12 (60.00)	12 (60.00)	1/4 (25.00)	2/4 (50.00)	0/3 (0.00)
2019-67	C	1	20	6 (30.00)	7 (35.00)	0/4 (0.00)	3/4 (75.00)	–
2019-68	C	1	20	8 (40.00)	8 (40.00)	1/6 (16.67)	4/6 (66.67)	1/2 (50.00)
2019-69	C	1	20	9 (45.00)	10 (50.00)	–	–	1/3 (33.33)
2019-70	C	2	40	30 (75.00)	30 (75.00)	4/6 (66.67)	4/6 (66.67)	3/6 (50.00)
2019-71	C	3	68	45 (66.18)	43 (63.24)	5/12 (41.67)	12/14 (85.71)	7/8 (87.50)
2019-72	B	2	40	0 (0.00)	2 (5.00)	0/12 (0.00)	–	–
2019-73	B	2	40	0 (0.00)	0 (0.00)	–	–	–
2019-74	B	2	40	0 (0.00)	1 (2.50)	–	–	–
2019-75	B	2	40	10 (25.00)	11 (27.50)	3/12 (25.00)	–	–
2019-76	B	2	40	0 (0.00)	5 (12.50)	0/12 (0.00)	–	–
2019-77	B	2	40	28 (70.00)	28 (70.00)	8/12 (66.67)	–	–
2019-78	B	2	40	4 (10.00)	3 (7.5)	2/8 (25.00)	–	–
2019-79	C	2	40	21 (52.20)	24 (60.00)	4/12 (33.33)	10/12 (83.33)	5/6 (83.33)
2019-80	C	4	80	18 (22.50)	27 (33.75)	2/16 (12.50)	6/20 (30.00)	10/12 (83.33)
Total 80		233	3,470	1,259 (36.28)	1,795 (51.73)	230/591 (38.92)	185/291 (63.57)	239/295 (81.02)

*A: Layer of Chicken farm*.

*B: Breeder of Chicken farm*.

*C: Broiler of Chicken farm*.

*D: Broiler of Turkey*.

*E: Exotic Broiler Chicken farm*.

*Exotic chickens = A+E Native chickens = B+C*.

### Epidemiological Surveillance of ChPV/TuPV in Different Commercial Chicken and Turkey Flocks

**Figure 2:**
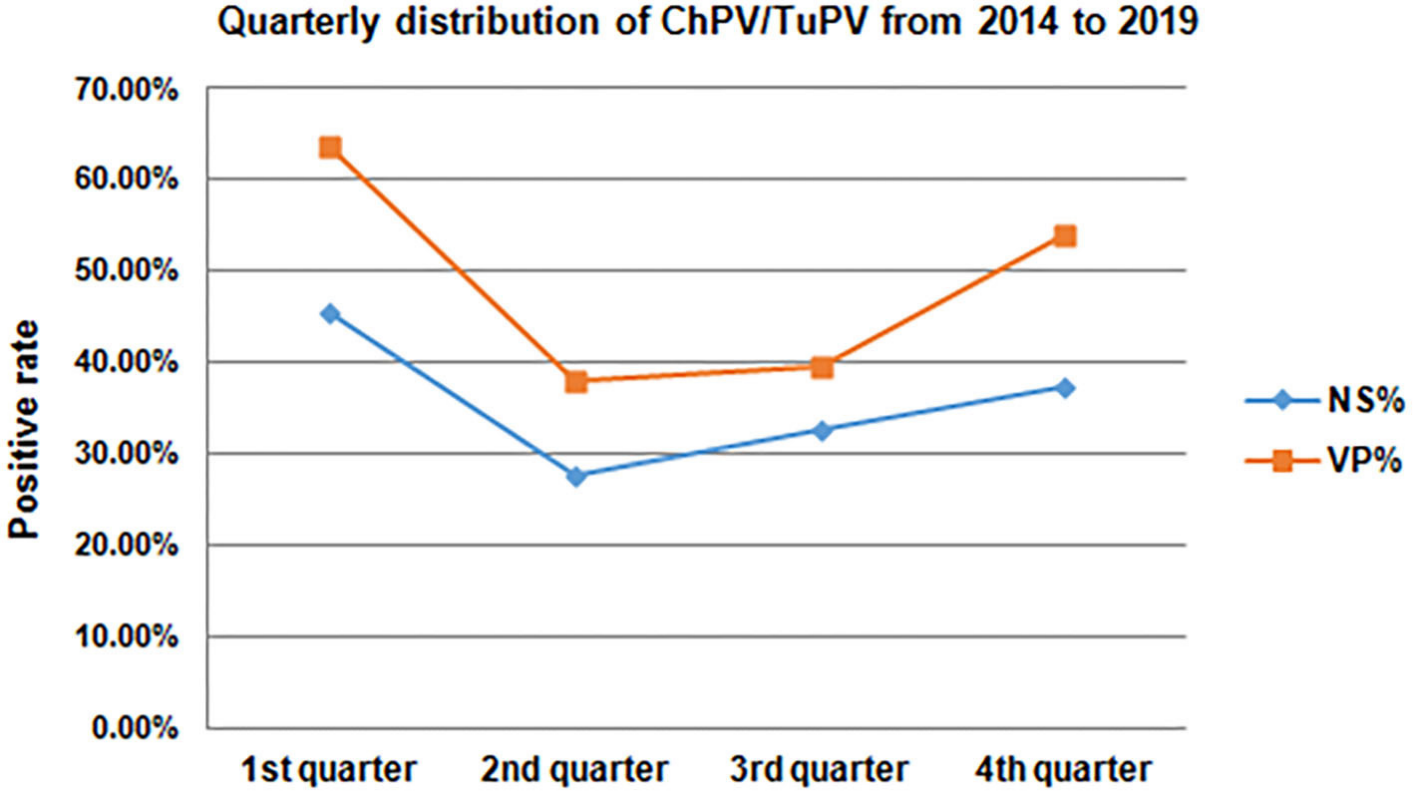
Quarterly distribution of ChPV/TuPV infections in chicken and turkey flocks from 2014 to 2019.

Of the 283 cloacal swabs collected from the 69 RSS-like flocks, 80.57% (228/283) were PCR-positive, while the 164 healthy chicken flocks had 49.17% (1,567/3,187) prevalence; of the 12 cloacal swabs collected from 1 PEMS-like flocks, 91.67% (11/12) were PCR-positive compared with the 5 healthy turkey flocks, which showed 81.94% (59/72) prevalence. Clinical samples from 227 chicken flocks (50.95%) aged 1–60 wks and 6 turkey flocks (83.33%) aged 3–47 wks were collected in 46 different counties in Guangxi. The highest frequency of ChPV positive samples tested by PCR occurred in chickens that were broiler chickens 64.18% (1,041/1,622) compared with breeder chickens 38.75% (572/1,476) and layer hens 38.89% (112/288) (Table 3). The prevalence of ChPV was higher in broiler chicks aged 1–7 wks (74.46%) than in birds in the 8–20 wk (53.56%) and over 21 wk (50.00%) age groups; in breeders aged 1–7 wks (74.12%) than in birds in the 8–20 wk (39.90%) and over 21 wk (17.28%) age groups; and in layer hens aged 3–28 wks (46.79%) than in birds in the 29–59 wk (32.50%) and 60–72 wk (0.00%) age groups; in turkey, the prevalence of TuPV was higher in the group aged 6–40 wks (100.00%) than in birds in the 0–5 wk (91.67%) and over 40 wk (0.00%) age groups. The highest frequency of ChPV positive samples tested by PCR occurred in chickens that were 1–7 wks of age, and TuPV was detected in 3–47 wk old turkeys. All seasons showed ChPV and TuPV circulation, especially from October to March (Figure 2). The presence of a parvoviral genome in samples was found in healthy poultry and in poultry suffering from RSS/PEMS symptoms. One to three samples from each farm were sequenced and submitted to GenBank (25).

**Table 3:** Percentage of positive samples for ChPV/TuPV according to chicken/turkey flock age.

**Broiler chickens**		**Breeders**		**Layer hens**		**Turkeys**	
**Wks**	**Number of positive samples**	**Wks**	**Number of positive samples**	**Wks**	**Number of positive samples**	**Wks**	**Number of positive samples**
1–7 w	624/838^a^ (74.46)^b^	1–7 w	295/398 (74.12)	3–28 w	73/156 (46.79)	0–5 w	22/24 (91.67)
8–20 w	376/702 (53.56)	8–20 w	160/401 (39.90)	29–59 w	39/120 (35.00)	6–20 w	24/24 (100.00)
≧21 w	41/82 (50.00)	≧21 w	117/677 (17.28)	60–72 w	0/12 (0.00)	20–40 w	24/24 (100.00)
						≧40 w	0/12 (0.00)
Total	1,041/1,622 (64.18)	572/1,476 (38.75)		112/288 (38.89)		70/84 (83.33)	

a *Positive/total number of ChPV/TuPV detected*.

b *Percentage of flocks positive for ChPV/TuPV*.

The prevalence rates of ChPV were significantly different in different sources of chickens (Table 4). All the layer hens belong to exotic chickens. Exotic chickens had a 88.10% (148/168) VP gene positive rate; native chickens had a 50.00% (1,465/2,930) VP gene positive rate.

**Table 4:** The results of different ages between exotic and local chickens by ChPV detection.

	**No. of samples**	**Age**	**ChPV/TuPV PCR**
Exotic broiler chickens	144	1–7 w	94.44^a^ (136/144)^b^
	0	8–20 w	–
	24	≧21 w	50.00 (12/24)
			88.10 (148/168)
Exotic layer hens	156	3–28 w	46.79 (73/156)
	120	29–59 w	32.50 (39/120)
	12	60–72 w	0.00 (0/12)
			38.89 (112/288)
Native chickens	1,092	1–7 w	73.31 (783/1,092)
	1,103	8–20 w	48.59 (536/1,103)
	735	≧21 w	19.86 (146/735)
			50.00 (1,465/2,930)

a *Percentage of flocks positive for ChPV/TuPV*.

b *Positive/total number of ChPV/TuPV detected*.

Poultry farming may introduce pathogens into the environment and food chain. A total of 874 environmental samples were collected from two different systems of bird houses (open house and closed house). A higher prevalence of 59.31% (223/376) ChPV positivity was seen in swab samples (Table 5) than in other sources of samples, in which litter samples showed 57.14% (104/182) positivity, and drinking water samples showed 27.85% (88/316) positivity. Samples from open house flocks had higher prevalence rates of ChPV than those of closed house flocks (Table 5), among which those from the open house showed 84.16% (85/101) positivity, those from litter showed 62.86% (44/70) positivity, and those from drinking water showed 50.00% (56/112) positivity, whereas those from the closed house litter were 53.57% (60/112), those from swabs were 50.18% (138/275), and those from drinking water were 15.69% (32/204). Overall, 47.05% (415/874) of environmental samples from poultry houses tested positive for ChPV and TuPV using PCR-based specific detection.

**Table 5:** The results of environmental sampling of chicken and turkey houses by ChPV/TuPV detection.

	**Closed houses**	**Open houses**	**Total**
Litter A	53.57 (60/112)	62.86^a^ (44/70)^b^	57.14 (104/182)
Water B	15.69 (32/204)	50.00 (56/112)	27.85 (88/316)
Swab C	50.18 (138/275)	84.16 (85/101)	59.31 (223/376)
Total	38.92 (230/591)	63.57 (185/291)	47.05 (415/882)

a *Percentage of flocks positive for ChPV/TuPV*.

b *Positive/total number of ChPV/TuPV detected*.

*Litter A: including floor and cleaning poultry wastewater*.

*Water B: including all kinds of drinking water in the henhouse*.

*Swab C: including egg cartons, coop, skip car, and chopping boar*.

Forty-six counties belonging to the 10 cities in Guangxi were investigated for the prevalence of ChPV/TuPV in farm, and the top five cities in terms of positive rates of ChPV/TuPV were Liuzhou, Yulin, Beihai, Nanning, and Wuzhou, with positive rates of 100, 67.5, 66.67, 61.09, and 60.42%, respectively.

## Discussion

ChPV is a widely prevalent pathogen and has global significance because it adversely affects poultry health and production systems by increasing morbidity, the ratio of feed to meat, and secondary infections. In the present study, specific clinical signs of ChPV were generally observed during the first 2–4 wks of age, when susceptible chicks were infected by vertical or horizontal routes of transmission. The positive incidence was higher and the age of poultry was lower in all kinds of chickens and turkeys in our study; it was believed that there was age-related development of resistance against ChPV due to immune competence. Exotic broiler chickens showed a higher prevalence of ChPV than Chinese native chickens, indicating the Chinese native chickens may have stronger immune systems. The low ChPV positive rate of exotic Layer hens was due to sick chickens being eliminated in the initial stage. However, subclinical infections usually occur in adult birds and were responsible for production losses and secondary infection. Subclinical infections also act as a source of infection for other young birds and flocks. Additionally, complex factors such as secondary bacterial, fungal, or viral infections may exacerbate the course of parvovirus infection. We used two published PCR assays to confirm the PCR-positive samples, and the results showed that the nPCR (249 bp) has a higher positive rate than the PCR (561 bp), which was used to detect ChPV/TuPV in most countries. It indicated that VP PCR was more sensitive under experimental conditions in our study. This result leads us to ask whether the prevalence of ChPV/TuPV in the world is more severe than previously thought?

The Guangxi Zhuang Autonomous Region is located in southern China. Guangxi borders Vietnam, where avian influenza is also prevalent and the epidemiology of zoonotic diseases is complex. There are several large-scale poultry farms and large numbers of small-scale farms in Guangxi. The booming poultry industry in the region faces a significant risk for RSS/PEMS, and there is little epidemiological surveillance of ChPV and TuPV in the region. The top five cities in terms of positive rates of ChPV/TuPV were Liuzhou, Yulin, Beihai, Nanning, and Wuzhou. Because these are the main regions of the poultry industry of broiler chickens in Guangxi, these findings indicate that the more commercial poultry farms there are in cities, the higher the ChPV/TuPV positivity rate. Moreover, a city's ChPV/TuPV positive rate is also associated with the transportation distance and the clinical sample collection method. All turkey samples were collected around Nanning. In addition, ChPV and TuPV were identified in each season. The region's warm and humid climate may facilitate the survival, growth and transmission of ChPV and TuPV, as well as the occurrence of mixed infections. The selections of poultry farms in this study was designed to cover different geographic areas in Guangxi, but samples from a few areas could not be obtained, meaning that our results were not fully representative. The possibility of some differences in the distribution of viral variants/genotypes due to sampling bias cannot be ruled out, especially for cities where the number of samples was low.

The detection of ChPV in poultry at 1 wk of age is not surprising, as published studies suggested the possibility of vertical viral transmission (26, 27). However, we demonstrated the absence of parvovirus in cloacal swabs from 1- to 5-day-old broiler chickens, which was different from Finkler's report (28), and this finding may be due to the different pathogenicity of the virus strains or the different chicken breeds. In our study, parvovirus was detected mainly in healthy birds. In fact, many chicken and turkey flocks that tested positive for parvovirus showed no signs of disease. This result is consistent with previous work by Zsak et al. although in another study, parvoviruses were detected in poultry with no enteric disorders (16, 20, 29). In addition, the parvovirus infection rate of turkeys appeared to be higher than that of chickens. The five turkey flocks which were highly positive for parvoviruses included both weak and healthy flocks.

In our study, 47.05% (415/882) of environmental samples from poultry houses tested positive for parvovirus, and a higher prevalence of parvovirus positives was seen in open house flocks' samples than closed house samples, which indicated that the biosecurity measures of farmers who raised the birds in closed houses were applied well. An important factor in RSS/PEMS epidemiology in poultry farms is the introduction of enteric viruses into a flock. Moreover, the persistence of viruses in a flock can influence the course of disease and viral spread or distribution to neighboring farms. Some cases of RSS/PEMS may be attributed to wild birds that are shedding the viruses. These animals begin latent viral excretion before clinical symptoms appear. Birds that have recovered from clinical infection may also be shedders. Birds incubating the virus can spread it to healthy, uninfected birds. Farmers in Guangxi have received little information about RSS and PEMS prevention. Birds move freely, there are no restrictions on human or vehicle movement, and biosecurity measures are not well-applied at many farms. Therefore, it is important to implement biosecurity measures and to control the movements of animals and instruments to minimize the spread of ChPV/TuPV shed by both healthy and sick birds, and, finally but importantly, to improve the nutrition of all the birds in commercial farms.

Considering the emerging status of parvovirus and its wide prevalence, recent advances in diagnosis, vaccinations and therapeutics, along with appropriate disease prevention and control strategies, need to be followed to curtail high losses in the poultry industry due to this economically important pathogen. Further investigations focusing on whether genomic recombination occurred during mixed infection are needed.

In conclusion, this report is the first ChPV and TuPV epidemiological survey to document the presence in Guangxi. Our study determined a prevalence of 51.73% of ChPV and TuPV in apparently healthy and RSS/PEMS-like commercial poultry farms. Our reseach has published an article comparing the molecular properties of ChPV and TuPV (25). Commercial poultry farms are very common in China, so we first focused on commercial farms. We are planning to conduct more tests for parvoviruses in various types of chicken farms including small non-commercial flocks for future study. Further extensive epidemiological studies are recommended to determine the true amount of ChPV and TuPV infection in poultry farms and to adopt timely prevention and control strategies, which would help alleviate the economic losses caused by this economically detrimental and emerging virus affecting the poultry industry.

## Data Availability Statement

All datasets generated for this study are included in the article/supplementary material.

## Ethics Statement

The animal study was reviewed and approved by the Animal Ethics Committee of the Guangxi Veterinary Research Institute.

## Author Contributions

ZhixX designed and coordinated the study and helped to review the manuscript. YZ and BF performed the experiments. XD, YZ, BF, ZhiqX, SL, QF, and SW were responsible for collecting the clinical samples. BF, YZ, LX, MZ, JH, and TZ analyzed the data. BF wrote the manuscript. YZ checked and edited the manuscript. All authors contributed to the article and approved the submitted version.

## Conflict of Interest

The authors declare that the research was conducted in the absence of any commercial or financial relationships that could be construed as a potential conflict of interest.
